# Bone volume, mineral density, and fracture risk after kidney transplantation

**DOI:** 10.1371/journal.pone.0261686

**Published:** 2022-03-29

**Authors:** Satu Keronen, Leena Martola, Patrik Finne, Inari S. Burton, Xiaoyu F. Tong, Heikki Kröger, Eero Honkanen

**Affiliations:** 1 Department of Nephrology, Abdominal Center, University of Helsinki and Helsinki University Hospital, Helsinki, Finland; 2 Kuopio Musculoskeletal Research Unit (KMRU), University of Eastern Finland, Kuopio, Finland; 3 Department of Orthopedics, Traumatology and Hand Surgery, Kuopio University Hospital, Kuopio, Finland; University of Liège, BELGIUM

## Abstract

**Background:**

Disordered mineral metabolism reverses incompletely after kidney transplantation in numerous patients. Post-transplantation bone disease is a combination of pre-existing chronic kidney disease and mineral disorder and often evolving osteoporosis. These two frequently overlapping conditions increase the risk of post-transplantation fractures.

**Material and methods:**

We studied the prevalence of low bone volume in bone biopsies obtained from kidney transplant recipients who were biopsied primarily due to the clinical suspicion of persistent hyperparathyroidism between 2000 and 2015 at the Hospital District of Helsinki and Uusimaa. Parameters of mineral metabolism, results of dual-energy x-ray absorptiometry scans, and the history of fractures were obtained concurrently.

One hundred nine bone biopsies taken at a median of 31 (interquartile range, IQR, 18–70) months after transplantation were included in statistical analysis. Bone turnover was classified as high in 78 (72%) and normal/low in 31 (28%) patients. The prevalence of low bone volume (n = 47, 43%) was higher among patients with low/normal turnover compared to patients with high turnover [18 (58%) vs. 29 (37%), P = 0.05]. Thirty-seven fragility fractures in 23 (21%) transplant recipients corresponding to fracture incidence 15 per 1000 person-years occurred during a median follow-up 9.1 (IQR, 6.3–12.1) years. Trabecular bone volume did not correlate with incident fractures. Accordingly, low bone mineral density at the lumbar spine correlated with low trabecular bone volume, but not with incident fractures. The cumulative corticosteroid dose was an important determinant of low bone volume, but not of incident fractures.

**Conclusions:**

Despite the high prevalence of trabecular bone loss among kidney transplant recipients, the number of fractures was limited. The lack of association between trabecular bone volume and fractures suggests that the bone cortical compartment and quality are important determinants of bone strength and post-transplantation fracture.

## Introduction

Bone volume, reflected by bone mineral density (BMD), as well as bone quality, contributes to bone strength which is altered in patients with chronic kidney disease (CKD). The risk of fractures increases with declining kidney function [1,2]. However, traditional risk factors of osteoporosis (e.g., increased age, diabetes, malnutrition, physical inactivity, hypogonadism, and smoking) account only partly for the excessive risk of fractures among the CKD population [3–5]. Bone turnover and mineralization, which are also important contributors to bone quality, are altered in almost all CKD patients.

In a large proportion of transplant recipients, pre-existing chronic kidney disease-mineral and bone disorder (CKD-MBD) reverses incompletely, especially with declined allograft function. Low bone formation due to immunosuppressive therapy, especially corticosteroids, further aggravates trabecular bone loss. These two often overlapping conditions increase the risk of post-transplantation fracture [6–11]. Besides the decreased quality of life, fractures increase the risk of hospitalization and mortality in transplant recipients [12].

Altered bone turnover is the primary target of the pharmacological treatment of CKD-MBD. Parathyroidectomy is considered in patients with hyperparathyroidism refractory to pharmacological treatment with vitamin D analogous either alone or combined with calcimimetics. However, antiresorptive or anabolic agents are required for the treatment of osteoporosis. Differentiation between these conditions is therefore necessary for the accurate treatment of the patient and to prevent consecutive fractures.

In the general population low bone volume and impaired mineralization are associated with an increased risk of fracture. The histomorphometric analysis of iliac crest bone biopsy has been selected as the most precise method to evaluate bone metabolism [13]. Bone biopsy, however, is infrequently performed due to its`invasive nature and sample analysis requiring specific expertise. Bone quantity can also be measured for the assessment of BMD using areal dual-energy x-ray absorptiometry (DXA). Growing evidence suggests the utility of decreased BMD to predict fractures also in transplant recipients [14–16].

This retrospective bone biopsy-based study was conducted to evaluate the prevalence of low bone volume and fractures after kidney transplantation. Another aim of this study was to analyze the relationship between bone histomorphometry, DXA, and fractures in kidney transplant recipients.

## Materials and methods

**Fig 1:**
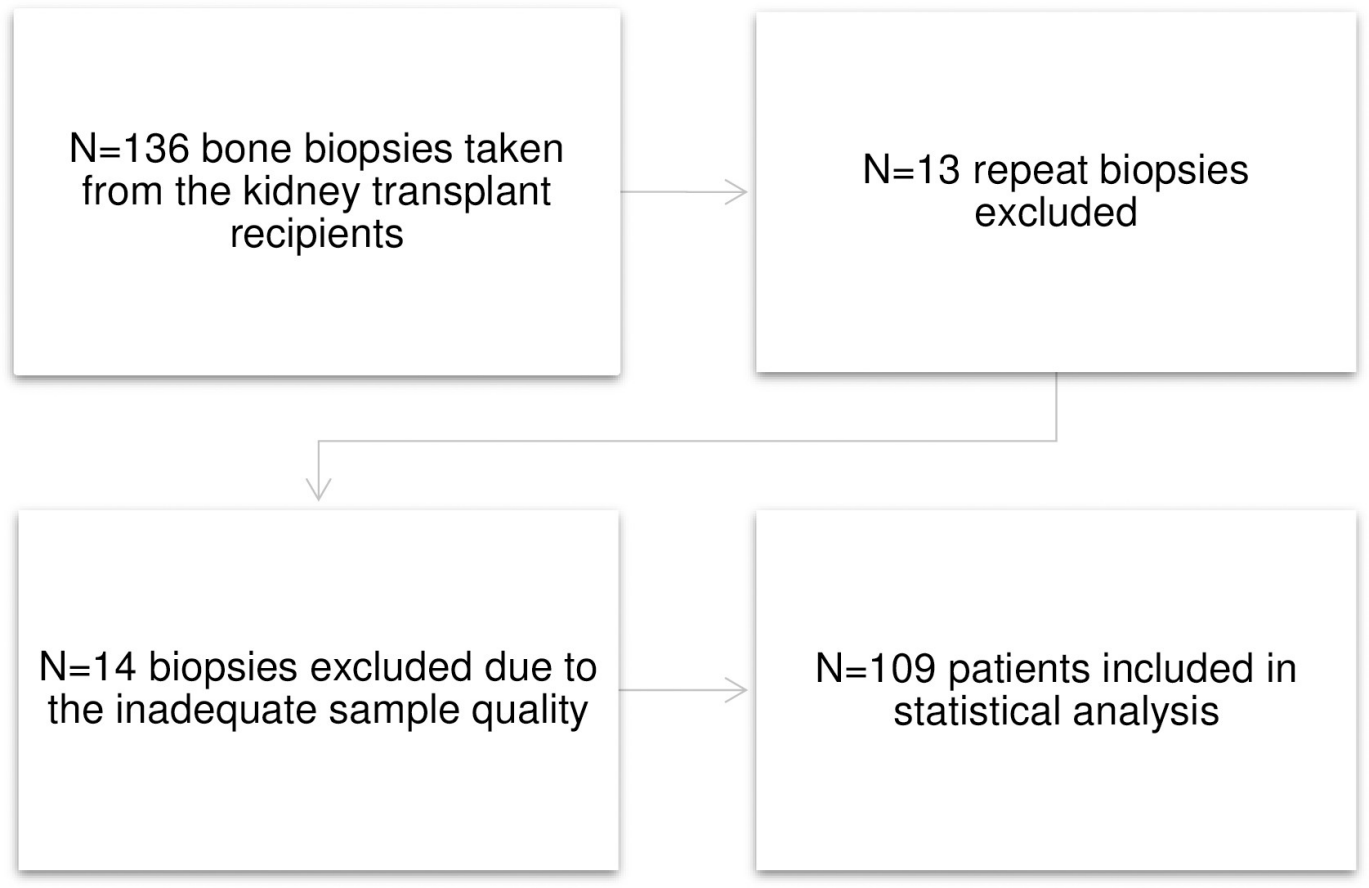
A flow chart of patient inclusion.

After obtaining approval from the Research Ethics Board of the Division of Medicine, Helsinki University Central Hospital (approval no. 413/13/03/00/09) and Institutional Review Board of the Hospital District of Helsinki and Uusimaa (HUS/33/2010, HUS/269/2017 and HUS/333/2019) with a waiver of informed consent of medical record review, the medical records of transplant recipients referred for bone biopsy between January 1, 2000, and December 31, 2015, were retrospectively screened. The flow chart of patients included in the study is presented in Fig 1. Thirteen repeat biopsies of 136 biopsies were excluded. The parameters of turnover and bone volume were determined in 109 patients, who were included in statistical analysis.

The clinical and research activities reported here are consistent with the Principles of the Declaration of Istanbul as outlined in the `Declaration of Istanbul on Organ Trafficking and Transplant Tourism´.

### Data collection

We reviewed electronic patient charts between September 30, 2019, and March 1, 2021, for relevant demographics (age, sex, medical comorbidities, fractures, previous parathyroidectomy, and mineral metabolism therapy at the time of biopsy) and laboratory findings. Data for prevalent (before transplantation) and incident fractures were collected from hospital records including surgery reports and documents of imaging examinations. Outpatient documents were not, however, available. Spine X-rays for screening asymptomatic vertebral fractures were not obtained. The localization and mechanism of injury were identified and fractures with documented prior trauma were excluded. The cohort entry date was the date of bone biopsy (between May 18, 2000, and October 5, 2015). Patients were followed until the return to maintenance dialysis, death, or the end of follow-up (December 31, 2019). The follow-up data varied between August 30, 2002, and December 31, 2019.

Plasma inorganic phosphate and ionized calcium, alkaline phosphatase (ALP), parathyroid hormone (PTH), plasma creatinine, and estimated glomerular filtration rate (eGFR) measured by the Chronic Kidney Disease Epidemiology Collaboration equation [17] were recorded at the time of or within three months preceding the bone biopsy. PTH at the time of transplantation was also available in a subset of patients. Plasma inorganic phosphate (reference range 0.71–1.41 mmol/l) was analyzed by photometric determination with Modular E170 analyzer (catalog number 1730347, Roche Diagnostics, Indianapolis, IN), while ionized calcium (reference range 1.16–1.3 mmol/l/pH 7.4) was analyzed by direct ion-selective electrode method with Radiometer ABL800 analyzer (Radiometer Medical). ALP was measured by enzyme-linked immunosorbent assay (BM systems ALP between 2000 and October2005 and since November2005 ALP IFCC liquid) with Modular E170 analyzer (Roche Diagnostics, Indianapolis, IN). The reference range was 60–275 U/l until April 28, 2004, and since April 29, 2004, 35–105 U/l. Between 1998 and May 14, 2000, serum intact PTH levels (reference range 15–60 ng/l) were studied by immunoradiometric assay (INTACT PTH, catalog number 40–2170, Nichols Institute Diagnostics, San Juan Capistrano, CA). LIAISON (DiaSorin, Stillwater, MN) analyzer with immunoradiometric assay by Nichols (reference range 15–60 ng/l) was used to study intact plasma PTH levels between May 15, 2000, and September 9, 2001. Immunochemiluminometric assay (Immulite 2000 intact PTH, catalog number L2KPP2, reference range 15–73 ng/l) and Immulite 2000 Systems analyzer (Siemens Healthcare Diagnostics) was used from September 10, 2001, to May 31, 2011. Since June 1, 2011, electrochemiluminescence immunoassay (reference range 12–47 ng/l) with Modular E170 analyzer (Roche Diagnostics, Indianapolis, IN) was used. Since January 15, 2014, the reference range for the same method was changed to 15–65 ng/l. All assays were performed according to manufacturers´ instructions at HUS-LAB, at Meilahti laboratory, Helsinki, Finland.

### Bone biopsy and histomorphometric analysis

Iliac crest bone biopsies were obtained 5–14 days after the second labeling with tetracycline (500 mg 3 times/day over two separate 2-day periods with a 10-day interlabel time) and under local anesthesia. Bone biopsied were obtained with a drill (Straumann, Switzerland) until the year 2005 and thereafter the vertical technique by 8G – 11G needle (T-Lok, Angiotech, Reading, PA, USA) was used.

The technique for quantitative histomorphometry has been described previously [18]. A semiautomatic image analyzer [Osteoplan II system (Carl Zeiss, Thornwood, NY) until the year 2004 and thereafter BioquantOsteoII (Bioquant Image Analysis Corporation, Nashville, TN, USA)] were used for performing histomorphometric analyses at standardized sites in the trabecular bone at x200 magnification.

Bone turnover was determined by the bone formation rate per bone surface (BFR/BS, normal reference value 18–38 μm3/μm2/year) and activation frequency (Ac.F, normal reference value 0.49–0.72/year) [19]. In the absence of tetracycline labeling, or if only a single label was found in the trabecular bone area, the assessment of bone turnover was made using osteoblastic (Ob.S/BS, %) and osteoclastic surfaces (Oc.S/BS, %). The reference values were applied as Z-scores based on Rehman et al. [20]. Mineralization was identified as abnormal when osteoid surface/bone surface (OS/BS, %) was more than ±2 SD compared with the mean value [19] and/or mineralization lag time (Mlt, days) exceeded 100 days [21]. The normal range of trabecular bone volume/tissue volume (BV/TV) was 16.8–22.9% [20]. The final classification of bone turnover and volume, however, was not based entirely on bone histomorphometric parameters, but on the consensus statement of two experienced histomorphometrists (HK, IB) also.

### Bone densitometry

DXA scans taken during the preceding 12 months of the bone biopsy were included, while scans taken following 12 months after biopsy were included only if no interventions were done after the biopsy.

Until the year 2009 Hologic QDR 4500W scanner (Hologic, Marlborough, MA) and thereafter Lunar Prodigy scanner (GE Healthcare, Little Chalfont, UK) were used for the measurements of BMD at the lumbar spine and femoral neck. The coefficients of variation for DXA measurements were at lumbar spine 1% and femoral neck 1.5%. The BMD values were given in g/cm^2^, and individual patient´s results were expressed as T-scores. Osteopenia was defined as a T-score between -1 and -2.5 and osteoporosis as a T-score -2.5 and below.

### Statistical analysis

The results were reported according to STROBE statement guidelines for observational studies. We divided bone biopsy findings into two groups according to bone volume (low or normal) for statistical analysis. Bone turnover and mineralization were determined according to turnover-mineralization-volume classification. To compare PTH values at different time points, we used the conversion equations y(LIAISON) = 1.13(IRMA) +9 (R = 0.98), y(IMMULITE2000) = 0.99*(LIAISON)-0.6 (R = 0.98) and y(Modular) = 0.52*(IMMULITE2000) +11 ng/l. To allow comparisons between ALP values at different time points, we converted levels of ALP taken between January 1, 2000, and 28 April 28, 2004, by using the conversion equation y = ALP (old)*0.48. We imputed nine ALP values using the k-nearest neighbor approach [22]. The variables used for imputation were sex, age, the time between transplantation and bone biopsy, dialysis vintage, previous parathyroidectomy, bone turnover class, and the levels of ionized calcium and PTH. In 12 patients with only plasma total calcium level available, we converted levels of total calcium to ionized Ca by multiplying with 0.52. To compare differences in parameters between volume and fracture groups, we used Mann-Whitney U-test and Chi-square test for continuous and categorical variables, respectively. Kendall’s tau correlation coefficient was applied to determine correlations between continuous variables [23]. We performed all analyses with SPSS for Windows (version 25, SPSS, Chicago, IL, USA). All values are presented as the median and interquartile range (IQR, 25–75 percentiles) and number with percentage for nominal data. Statistical significance was defined as a two-sided P-value lower than 0.05.

## Results

### The characteristics of transplant recipients

In total, 109 biopsies (>95% white) were included in the statistical analysis. The indications for bone biopsy were hypercalcemia combined with elevated PTH levels in 68 (62%) patients and normocalcemia with elevated PTH levels in 38 (35%) patients, respectively. In one patient biopsy was obtained due to multiple fractures and in two patients due to isolated hypercalcemia.

The diagnosis of kidney disease was diabetic nephropathy in 23 (21%), polycystic kidney disease in 20 (18%), glomerulonephritis in 28 (26%), tubulointerstitial disease in 7 (6%), and hypertension/vascular in 4 (4%) patients. Twenty-seven (25%) patients had miscellaneous/other diseases.

Biopsies were taken at a median of 31 (, IQR, 18–70) months after kidney transplantation. The median follow-up time was 9.1 (IQR, 6.3–12.1) years.

No data on hypogonadism was available. However, considering the median age of transplant recipients (53, IQR, 46–62 years) and that menopause is documented to occur 5 years earlier among women with advanced kidney failure compared to the general population [24], we presumed that most women included in this study were postmenopausal.

### The characteristics of transplant recipients with low bone volume

**Fig 2:**
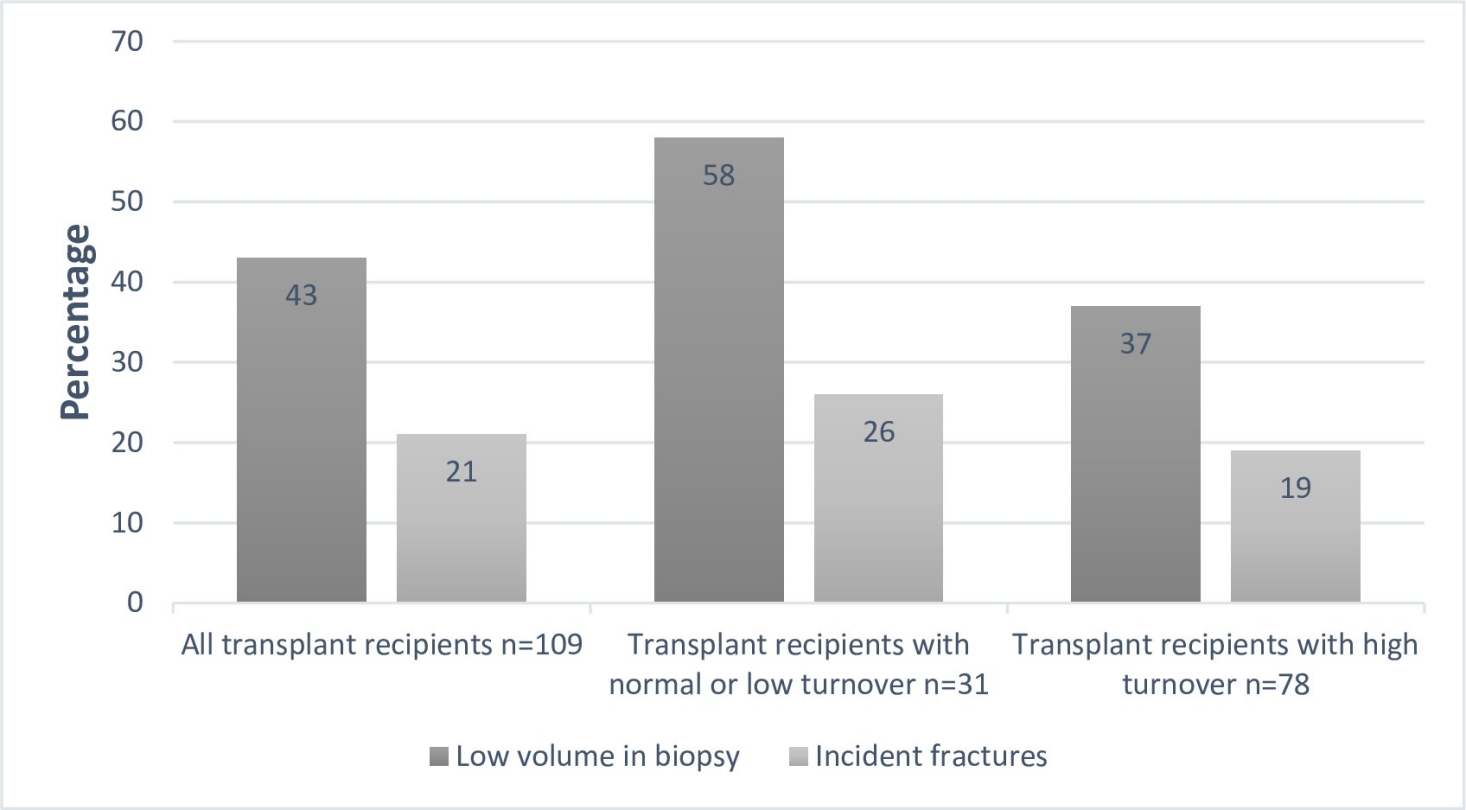
The distribution of low bone volume and fractures in transplant recipient groups.

The distribution of low bone volume and fractures is displayed in Fig 2. Bone volume was low in 47 (43%) transplant recipients. Bone turnover was classified as high in 78 (72%) and normal/low in 31 (28%) patients. The prevalence of low bone volume was higher among patients with low/normal turnover compared to patients with high turnover [18 (58%) vs. 29 (37%), P = 0.05]. The proportion of patients with impaired mineralization (19%) was similar in both volume groups.

The characteristics and details of mineral metabolism therapy of transplant patients with or without low bone volume in the bone biopsy are shown in Table 1. The maintenance immunosuppressive therapy comprised a calcineurin inhibitor, corticosteroids, and an antimetabolite. Median age, sex distribution (40% women), and the proportion of patients with diabetes (39%) were similar between volume groups. Thirty-six percent of patients were using bisphosphonates, with a median duration of 14 (IQR, 9–31) months preceding the biopsy. Patients with low bone volume had more coronary and peripheral artery disease compared to patients with normal bone volume (26% vs. 8%, P = 0.02 and 21% vs. 5%, P = 0.02, respectively). Compared to patients with normal volume, the cumulative corticosteroid exposure was higher in patients with low bone volume [5351 (IQR, 2694–10 972) mg in low volume group vs. 2704 (IQR, 2209–6178) mg in the normal volume group, P = 0.02]. Among patients with low bone volume bone biopsy was taken almost two years later than in patients with normal volume [45 (IQR, 21–80) months vs. 24 (IQR, 17–52) months, P = 0.03]. The use of bisphosphonates was similar among different volume groups.

**Table 1:** Characteristics of the transplant patients with or without low bone volume in bone biopsy.

Characteristic	All transplant recipients n = 109	Recipients with low bone volume n = 47	Recipients with normal bone volume n = 62	P-value
Female	44(40)	17(36)	27(44)	0.56
Age (years)	53(46–62)	56(46–65)	52(45–59)	0.13
Diabetes mellitus	42(39)	22(47)	20(32)	0.16
Dialysis vintage before biopsy (months) n = 106	25(13–43)	23(11–39)	27(16–52)	0.07
Timing of bone biopsy from KTX (months) n = 108	31(18–70)	45(21–80)	24(17–52)	**0.02**
Body mass index, (kg/m^2^)	25(22–27)	24(22–27)	25(22–28)	0.54
Coronary artery disease	17(16)	12(26)	5(8)	**0.02**
Peripheral artery disease	13(12)	10(21)	3(5)	**0.02**
Smoking n = 107	42(39)	14(30)	28(45)	0.12
Previous KTX	12(11)	4 (9)	8(13)	0.55
Previous PTX	9(8)	3(6)	6(10)	0.73
**Patients with incident (follow-up) fractures**	23(21)	13(28)	10(16)	0.14
**Corticosteroid use**	107(98)	46(98)	61(98)	1.00
Cumulative corticosteroid dose (mg) n = 106	3091(2253–8794)	5351(2649–10 972)	2704(2209–6178)	**0.02**
Bisphosphonate use	39(36)	19(40)	20(32)	0.42
Cinacalcet use	7(6)	1(2)	6(10)	0.14

Values are expressed as median + interquartile range (in parentheses) or number of patients (percentages in parentheses).

KTX, kidney transplantation; PTX, parathyroidectomy.

Laboratory values at the time of biopsy are displayed in Table 2. The kidney graft function was similar between volume and turnover groups with a median eGFR of 55ml/min (IQR, 45–72 ml/min). Median pre-transplantation PTH 265 (IQR, 169–421) ng/L did not differ between volume groups. Pre-biopsy PTH levels, however, were lower in patients with low bone volume compared to patients with normal bone volume [median PTH 126 (IQR, 96–184) ng/L, 105 vs. 147 ng/L P = 0.001].

**Table 2:** Biochemical parameters in transplant recipients with and without low volume.

Variable (reference range)	All transplant recipients n = 109	Transplant recipients with low volume n = 47	Transplant recipients with normal volume n = 62	P-value
eGFR ml/min (CKD-EPI)	55(45–72)	57(47–70)	54(44–74)	0.7
Pre-KTX PTH ng/l (15–65) n = 80	265(169–421)	247(174–337)	308(165–451)	0.27
Pre-biopsy PTH ng/l (15–65)	126(96–184)	105(83–151)	147(110–190)	**0.001**
Ca 2+ mmol/l (1.15–1.30)	1.32(1.26–1.37)	1.33(1.28–1.38)	1.31(1.25–1.37)	0.3
ALP U/l (35–105)	88(68–128)	84(64–122)	94(76–135)	0.15

Values are expressed as median + interquartile range (in parentheses). eGFR, glomerular filtration rate; CKD-EPI, Chronic Kidney Disease Epidemiology Collaboration; KTX, kidney transplantation; PTH, parathyroid hormone; Ca2+, ionized calcium; ALP, alkaline phosphatase.

### Prevalent and incident fractures

The characteristics, biochemical, and bone histomorphometric as well as densitometry findings in patients with or without fractures are displayed in Table 3.

**Table 3:** Characteristics, biochemical parameters, bone histomorphometry and densitometry findings in transplant recipients with and without incident fractures.

Variable (reference range)	All transplant recipients n = 109	Transplant recipients with fractures n = 23	Transplant recipients without fracture n = 86	P-value
Female (%)	44(40)	7(30)	37(43)	0.34
Age (years)	53(46–62)	48(39–54)	55(46–63)	**0.005**
Smoking (%)	42(39)	11(48)	31(36)	0.35
Body mass index (kg/m^2^)	25(22–27)	26(23–29)	24(22–27)	0.24
Cumulative corticosteroid dose mg n = 106	3091(2253–8794)	6178(2452–11713)	3006(2238–8200)	0.21
Timing of bone biopsy from KTX (months) n = 108	31(18–70)	32(20–74)	30(17–68)	0.35
Previous PTX	9 (8)	2 (9)	7 (8)	1.0
PTX after bone biopsy	35(32)	7(30)	28(33)	1.0
Pre-KTX PTH ng/l (15–65) n = 80	265(169–421)	177(154–259)	326(186–450)	**0.007**
Pre-biopsy PTH ng/l (15–65)	126(96–184)	108(79–180)	132(101–185)	0.24
Ca 2+ mmol/l (1.15–1.30)	1.32(1.26–1.37)	1.31(1.23–1.34)	1.33(1.27–1.38)	0.06
**ALP U/l (35–105)**	88(69–129)	91(77–129)	85(67–128)	0.57
High bone turnover (%)	78(72)	15(65)	63(73)	0.45
BV/TV%	20.1(14.5–26.3)	18.1(16–25.8)	20.4(13.5–26.7)	0.83
BMD g/cm^2^ FN n = 54	0.69(0.61–0.736)	0.724(0.562–0.777)	0.667(0.609–0.73)	0.42
T-score in FN n = 54	-1.9 (-2.5 to -1.3)	-1.7 (-2.8 to -1.2)	-2.0 (-2.6 to -1.4)	0.57
BMD cm^2^ LS n = 54	0.915(0.856–1.052)	0.933(0.894–1.069)	0.90(0.819–1.054)	0.25
T-score in LS n = 55	-1.4(-2.0 to -0.5)	-1.3(-1.6 to -0.7)	-1.7(-2.2 to -0.5)	0.26

Values are expressed as median + interquartile range (in parentheses). KTX, kidney transplantation; PTH, parathyroid hormone; PTX, parathyroidectomy; Ca2+, ionized calcium; ALP, alkaline phosphatase; BV/TV, bone volume/tissue volume; BMD, bone mineral density; FN, femoral neck; LS, lumbar spine.

Four patients experienced a fracture before kidney transplantation. During the follow-up time, 37 fragility fractures occurred in 23 (21%) transplant recipients corresponding to fracture incidence of 15 per 1000 person-years. Eight (7%) patients experienced multiple fractures. One fracture was vertebral and 36 fractures were non-vertebral (hip 2, rib 4, leg 13 arm 14, and ankle 3).

Median time to the first fracture after transplantation was 7 (IQR, 1–12) months. Patients with fractures were a median of seven years younger than patients who did not experience a fracture. Neither gender, diabetes, BMI, smoking, dialysis vintage, the timing of the biopsy after transplantation, use of bisphosphonates nor history of the previous parathyroidectomy correlated with fractures. The cumulative corticosteroid exposure did not differ between patients with fractures or those without them (6178 mg vs. 3006 mg, P = 0.21). Median pre-transplantation PTH was, however, lower in patients with fractures compared to patients without them (177 vs. 326 ng/L, P = 0.007). Neither pre-biopsy PTH nor ALP levels correlated with fractures.

### Bone histomorphometric parameters and fractures

Bone histomorphometric parameters among transplant recipients with or without low bone volume are shown in Table 4. Tetracycline labeling was found in 99 (91%) bone biopsies. Either Ob.S/BS, Oc.S/BS, or BFR were available in all included biopsies.

**Table 4:** Bone histomorphometric parameters in transplant recipients with and without low volume.

Variable	All transplant recipients n = 109	Transplant recipients with low volume n = 47	Transplant recipients with normal volume n = 62	P-value
Bone formation rate/bone surface μm3/μm2/year n = 67	16.79(7.30–29.20)	10.73(6.53–21.90)a	21.9(10.95–42.24)b	**0.005**
Activation frequency (1/yr) n = 55	0.40(0.27–0.87)	0.31(0.15–0.52)c	0.64(0.27–1.00)d	**0.01**
**Osteoid surface/bone surface %**	31.80(21.90–47.90)	31.80(22.70–41.70)	32.6(21.66–52.89)	0.35
**Osteoblastic surface/bone %**	2.90(1.40–6.50)	2.46(1.40–5.60)	3.7(1.46–8.01)	0.15
**Osteoclastic surface/bone %**	1.17(0–3.17)	0.25(0–1.80)	1.94(0–3.71)	**0.005**
Osteoid thickness μm	8.4(6.6–11)	7.30(6.1–10.6)	9.2(6.7–11.7)	**0.05**
Mineralization lag time days n = 59	62.4(45.4–93.8)	59.9(37.8–94.7)e	62.8(45.6–93.8)f	0.71
Bone volume/tissue volume %	20.1(14.5–26.3)	13.2(10.5–16.6)	25.6(21–30.5)	**<0.001**

Values are expressed as median + interquartile range (in parentheses). ^a^n = 22; ^b^n = 45; ^c^n = 17; ^d^n = 38; ^e^n = 20; ^f^n = 39.

The distribution of bone turnover did not differ between patients with fractures and those without them. Accordingly, the proportion of patients with abnormal mineralization was similar in patients with fractures compared to those without them (22% vs. 19%).

The level of BV/TV did not differ between patients with fractures and those without them [18.1 (IQR, 16–25.8) % with fractures vs. 20.4 (IQR, 13.5–26.7) % without fractures, P = 0.47]. Ten (16%) patients with normal bone volume and 13 (28%) patients with low bone volume in bone biopsy experienced a fracture, but the difference did not reach statistical significance.

### Bone mineral density

DXA measurements at the lumbar spine and femoral neck were available in 54 (50%) patients. Accordingly, DXA was available in 17 (74%) patients with fractures, respectively. DXA scan was obtained a median of two (IQR, nine months before to six months after) months before the biopsy. The use of bisphosphonates was more common in the DXA group (69% with DXA vs. 31% without DXA, respectively), but otherwise the characteristics or laboratory values of patients with DXA scan did not differ from patients without DXA scan.

The results of DXA measurements among transplant recipients with or without low bone volume are shown in Table 5.

**Table 5:** Dual-energy x-ray absorptiometry measurements in transplant patients with or without low bone volume.

Variable	All transplant recipients n = 109	Transplant recipients with low volume n = 47	Transplant recipients with normal volume n = 62	P-value
Femoral neck T-score n = 54	-1.9(-2.5-to -1.3)	-2.2(-2.8 to -1.6)^a^	-1.5(-2.4 to -1.1)^b^	0.06
Femoral neck BMD g/cm^2^ n = 54	0.69(0.61–0.736)	0.667(0.604–0.728)	0.715(0.621–0.749)	0.25
**Lumbar spine T-score n = 55**	-1.4(-2.0 to -0.5)	-1.8(-2.5 to -1.2)^c^	-1.1(-1.63 to -0.2)^d^	**0.006**
**Lumbar spine BMD g/cm**^**2**^ **n = 54**	0.915(0.856–1.052)	0.887(0.789–0.942)	0.946(0.884–1.095)	**0.01**

Values are expressed as median + interquartile range (in parentheses). ^a^n = 28; ^b^n = 26; ^c^n = 29; ^d^n = 26.

BMD, bone mineral density.

BMD was significantly lower at the lumbar spine among patients with low bone volume in the biopsy, but no difference was found at the femoral neck.

Neither lumbar spine nor femoral neck BMD differed between patients with fractures compared to those without them (at lumbar spine 0.933g/cm^2^ vs. 0.900 g/cm^2^, P = 0.25 and at femoral neck 0.724g/ cm^2^ vs. 0.667g/cm^2^, P = 0.42).

**Fig 3:**
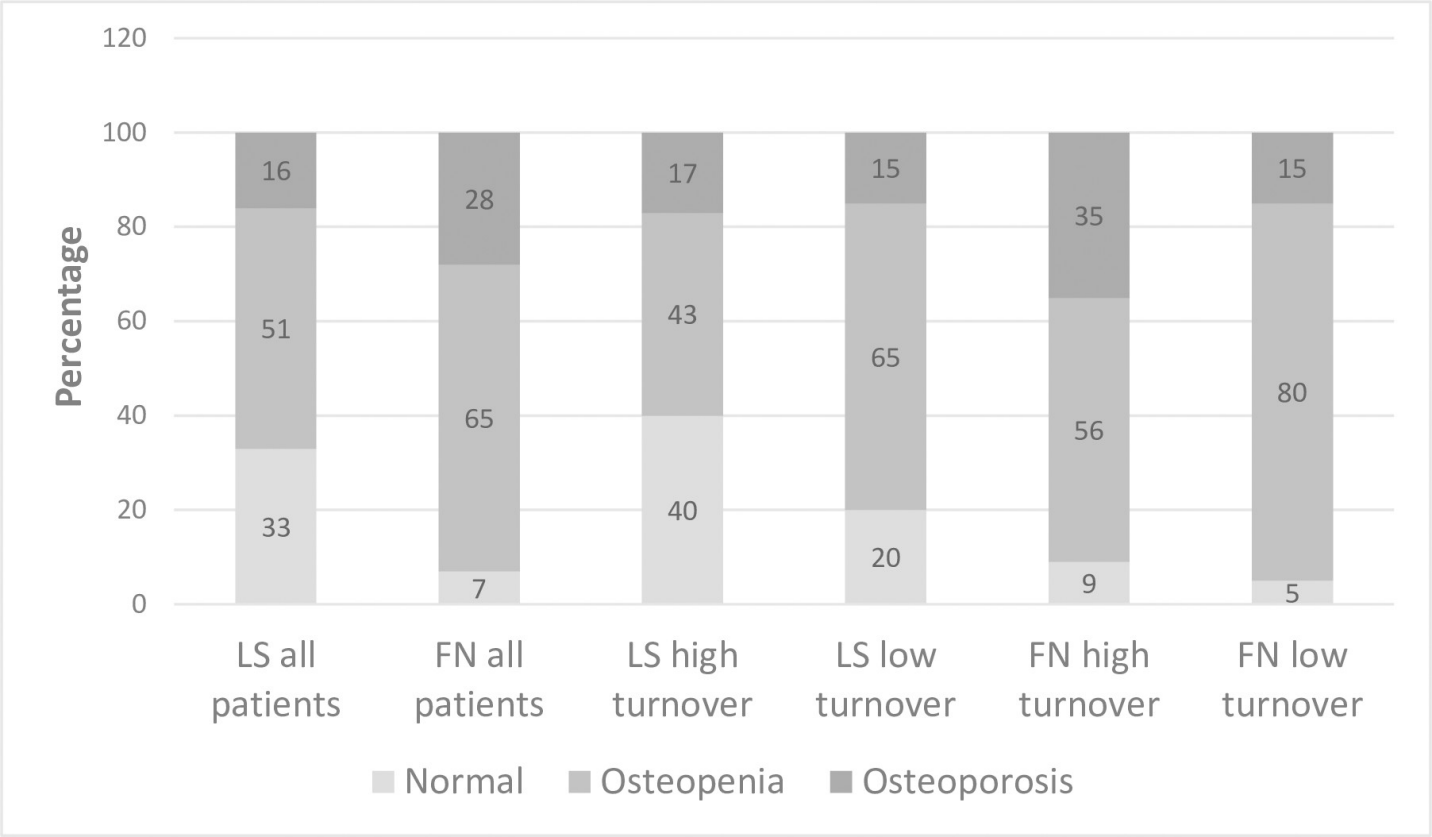
The prevalence of osteopenia and osteoporosis at the lumbar spine and femoral neck in DXA scan.

**Fig 4:**
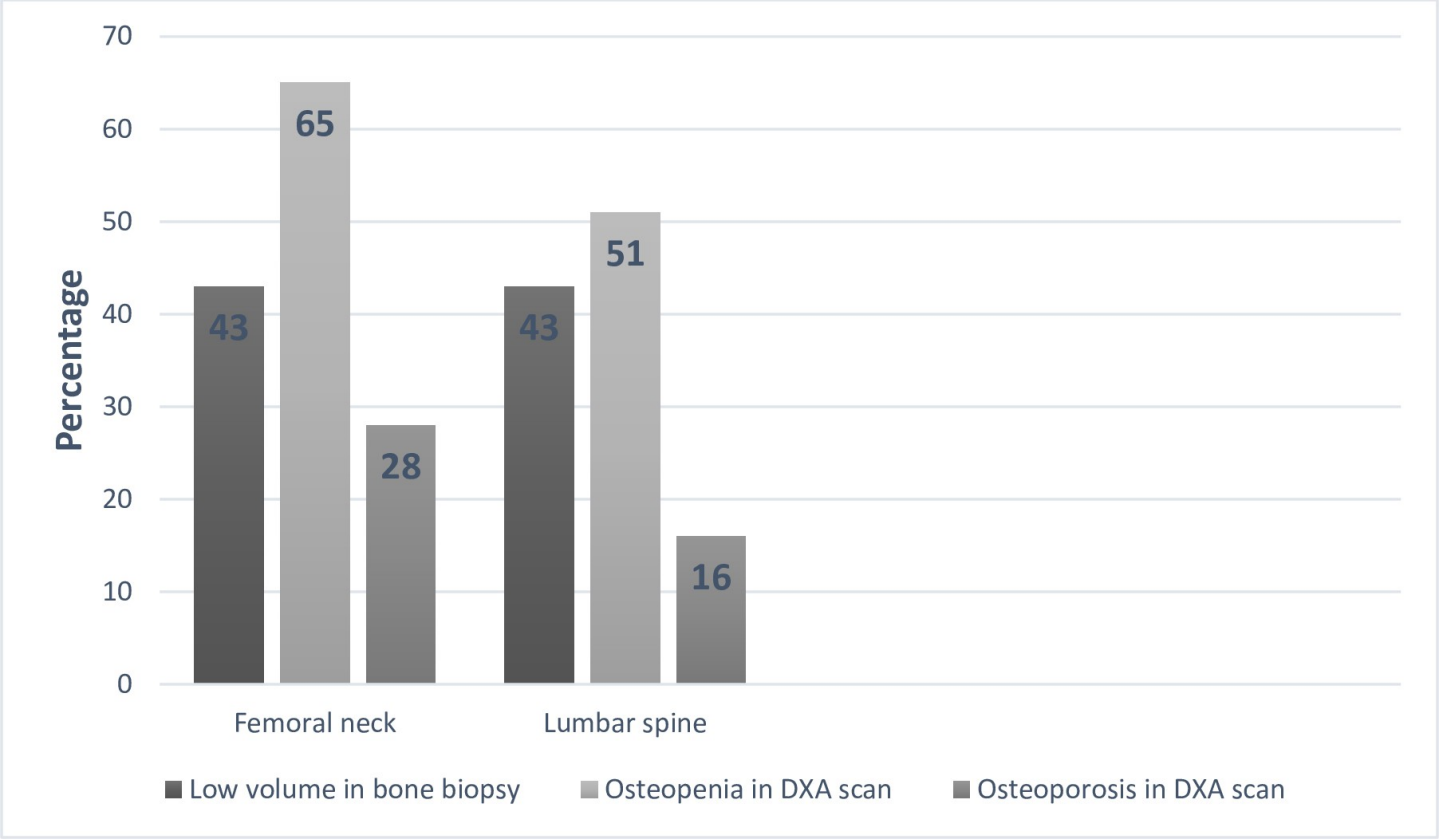
The diagnostic overlap of low bone volume on bone biopsy vs. osteoporosis in DXA scan.

The prevalence of osteoporosis and osteopenia at the lumbar spine and femoral neck according to World Health Organization criteria for DXA is displayed in Fig 3. The diagnostic overlap of low bone volume on the iliac bone biopsy and DXA scans is shown in Fig 4.

## Discussion

In this bone biopsy-based study including mainly patients biopsied due to persistently elevated PTH levels, the prevalence of low bone volume was 43%. Applying WHO criteria for DXA scans, osteoporosis was present at different skeletal sites in 16–28% of the study population, while osteopenia was detected in 51–65% of the patients. During the median follow-up time of 9.1 years, 23 (21%) transplant recipients sustained fragility fractures corresponding to fracture incidence of 15 per 1000 person-years. Low volume in the bone biopsy was associated with coronary or peripheral artery disease but had no association with low bone volume detected in the bone biopsy, but did not associate with fractures. In the low bone volume group, the cumulative corticosteroid exposure was significantly higher compared to the normal volume group, at least partially due to the significantly later timing of bone biopsy from kidney transplantation.

In previous bone biopsy-based studies [25–31] in transplant recipients, the prevalence of low volume has ranged between 11% and 63%. Although the prevalence of low bone volume in this study is in agreement with earlier studies, they are poorly comparable primarily due to the significantly higher proportion of patients with high turnover in our study. Besides turnover, the wide variation in patient characteristics, the cumulative corticosteroid exposure, the proportion of patients using bisphosphonates, and the timing of the assessment of bone biopsy after transplantation differ significantly between studies. As previously has been noted [32,33], the cumulative corticosteroid dose affects inversely to bone volume.

The proportions of osteoporosis and osteopenia in DXA scans are consistent with data reported in preceding studies [14,15] despite notable differences in case mix and timing of DXA after kidney transplantation. In contrast to these previous studies, however, we could not find an association between DXA and fractures. This difference may be explained by the insufficient power due to the small number of DXA scans. Another possible explanation is the high proportion of patients with persistent hyperparathyroidism. According to the literature, the ability of BMD to predict fractures may vary across different levels of PTH [15,34]. Correlations between bone trabecular volume and DXA parameters gave inconsistent results. This is hardly surprising since DXA is a combined composite of both trabecular and cortical bone volume. Another possible explanation for the low correlation between bone volume and DXA is a variation in trabecular bone mass and microarchitecture of the iliac crest. As recently suggested by European Consensus Statement on the diagnosis and management of osteoporosis in chronic kidney disease stages G4-G5D [16], a widely adopted osteoporosis intervention threshold (-2.5) in BMD may be too low for patients with advanced CKD. The high prevalence of osteopenia in this study suggests that a higher BMD threshold may be more appropriate also for transplant recipients.

In consonance with previous studies, fractures in this study were predominantly peripheral [35]. The number of fractures in our study is substantially lower than in earlier reports [9] but is in agreement with studies in more recent cohorts [10,11,14,15]. In the general population, the estimated yearly incidence of fractures in Finland is 0.63%. As previously reported in the general population, also patients with normal bone volume experienced fractures in this study. The lack of correlation with BV/TV and fractures suggests that the pathogenesis of fracture after transplantation is complex and not associated entirely with bone volume. The impaired bone quality also increases the risk of fracture [2,36]. In the general population, decreased cortical thickness and increased porosity have been associated with increased fracture risk. In CKD patients data of the cortical component of bone is, however, limited [37–39]. In the study by Carvalho et al. [28], compared to the trabecular component, cortical bone was less affected by post-transplantation changes of mineral metabolism. In the course of the present study, however, the cortical component was not analyzed.

Bone turnover is also a significant contributor to bone strength. In CKD patients, both low and high turnover has been shown to associate with fracture risk [40]. The importance of bone turnover as a risk factor of fracture after kidney transplantation is somewhat ambiguous. In this study, lower PTH levels at the time of transplantation, perhaps implying low bone turnover, were associated with post-transplantation fractures. The distribution of turnover in this study was, however, similar in patients who experienced a fracture. The cumulative corticosteroid dose, previously shown to decrease bone formation and density, did not associate with fractures. The relatively short interval between the first fracture and transplantation may explain the lack of this association. It is clinically noteworthy, that in this study parathyroidectomy was done to almost half of the patients with verified high turnover. Nonetheless, our study design does not allow causalities to be established between post-transplantation parathyroidectomy and incident fractures. Although cinacalcet has been shown to increase BMD in transplant recipients [41], its role in preventing fractures after transplantation is unknown. However, in this study, the number of patients using cinacalcet was too limited to make definite conclusions.

Although there is variation in observation time and site of fractures included, putting our results into the context of existing literature, there is a general downward trend in the risk of post-transplantation fractures. The most plausible explanations for this favorable outcome are changes in immunosuppressive therapy, especially the use of steroid-sparing protocols, and restraining from excessive suppression of hyperparathyroidism during the maintenance dialysis.

Several strengths and limitations must be addressed. The main strength of this study is the availability of bone histomorphometric data combined with mineral metabolism parameters and data on previous and incident fractures in a substantial number of kidney transplant recipients. We were not able to find other histomorphometric studies in transplant recipients where bone fractures were used as an endpoint. In addition to the highly selected patient cohort, the absence of the analysis of cortical porosity, mainly mediated by increased bone turnover [36], may explain the lack of association between trabecular bone volume and fractures. Fractures were identified from hospital records and documents of imaging examinations, but outpatient documents were not available. It is thus possible that data on especially peripheral fractures is incomplete. In addition, it is plausible that data on previous asymptomatic vertebral fractures is lacking, because systematic screening of lumbar X-rays was not performed. During the study period, the levels of calcidiol were not systemically evaluated after kidney transplantation, thus the role of vitamin D deficiency either in the prevalence of low bone volume or fracture rate cannot be estimated. Comprehensive data on gonadal status as well as post-transplantation metabolic acidosis was missing. Despite these limitations, however, the high prevalence of trabecular bone loss in conjunction with DXA scans showing predominantly decreased BMD confirms the deterioration of bone volume after kidney transplantation.

The generalizability of the observations of the high prevalence of low bone volume but a limited number of fractures after kidney transplantation is hampered by the high percentage of patients with high bone turnover and identical ethnic backgrounds. In addition, the high proportion of patients with post-transplantation parathyroidectomy may affect the number of incident fractures. Despite the even distribution of fractures in different bone turnover groups, the presented results cannot be extrapolated to all transplant recipients.

To conclude, post-transplantation bone loss affects a great proportion of kidney transplant recipients. Although according to the literature, the number of post-transplantation fractures has declined significantly during the past decade, the incidence of fractures is still substantial compared to the general population. However, as stated by Ott [42], a bone biopsy is not presumably the best measure of bone volume, although it remains a necessary tool for the determination of bone turnover and mineralization [43]. The lack of association between bone trabecular volume and fractures warrants the need for further studies to evaluate the role of bone microarchitecture and bone cortical component in fractures of kidney transplant recipients.

## Supporting information

S1 STROBE checklist Checklist of items that should be included in reports of *cohort studies*. (DOCX)
